# Effects of Pentoxifylline on Oxygenation and Exercise Tolerance in Patients with Severe Chronic Obstructive Pulmonary Disease

**Published:** 2013-06

**Authors:** Mohammad Javad Fallahi, Seiyed Mohammad Ali Ghayumi, Ali Reza Moarref

**Affiliations:** 1 Department of Internal Medicine, Nemazee Hospital, Shiraz University of Medical Sciences, Shiraz, Iran;; 2 Cardiovascular Research Center, Faghihi Hospital, Shiraz University of Medical Sciences, Shiraz, Iran

**Keywords:** COPD, Oxygenation, Pentoxifylline

## Abstract

**Background:** It was hypothesized that the use of Pentoxifylline would increase arterial O2 saturation and increase exercise tolerance in patients with Chronic Obstructive Pulmonary Disease (COPD).

**Methods: **We tested this hypothesis in 23 patients with COPD and pulmonary hypertension. Patients were randomized to receive Pentoxifylline or placebo, each for a 12-week period, in a prospective, double-blind study to assess the effects of Pentoxifylline on oxygen saturation and exercise tolerance via pulse oximetry and the 6-Minute Walk Test (6MWT).

**Results: **At the end of the 12 weeks, the six-minute walk distance rose from 351.9±65 meters to 393±67 meters in the Pentoxifylline group (10 patients) and increased from 328±79 meters to 353±66 meters in the placebo group (10 patients) (P=0.142). Resting oxygen saturation by pulse oximetry changed from 87±4% to 85±14% in the Pentoxifylline group and from 88±3% to 88±2% in the placebo group (P=0.676). There were no significant changes in dyspnea severity index and heart rate before and after the 6MWT.

**Conclusion:** Pentoxifylline does not seem to improve exercise capacity and dyspnea in patients with severe and very severe COPD.

## Introduction

Pentoxifylline is a methylxanthine and possesses several properties that could have beneficial effects for patients with Chronic Obstructive Pulmonary Disease (COPD) and pulmonary hypertension.^1^^-^^4^ With its anti-inflammatory, antifibrotic, and hemorheological properties,^5^ Pentoxifylline has been demonstrated to increase the filterability of red blood cells (RBCs), decrease the adherence of RBCs to endothelial cells, blood viscosity, platelet aggregation, fibrinogen levels, and act as a vasodilator and improve pulmonary hemodynamics.^6^^-^^11^These effects can reduce the incidence of hypoxia by improving blood delivery to vascular beds.^12^ In animal models, the beneficial effects of Pentoxifylline have been reported on hypoxia-induced skeletal muscle, lung, papillary muscle, and liver dysfunction.^13^^-^^17^ Furthermore, it is an effective adjunct to compression bandaging for treating venous ulcers and may decrease proteinuria in patients with diabetic nephropathy.^7^ The Food and Drug Administration (FDA) has approved its use for the management of intermittent claudication.^2^ It is deserving of note that the majority of adverse effects of Pentoxifylline are known to be gastrointestinal disturbances.^18^ There are, however, controversies over the beneficial effects of Pentoxifylline in patients with COPD with respect to improvement in the treadmill walk time, oxygen saturation, and dyspnea. In the past, a few studies demonstrated some beneficial effects of Pentoxifylline on pulmonary hypertension and pulmonary gas exchange.^1^^,^^3^^,^^4^ In contrast, Scott et al.^19^ failed to show any benefits of Pentoxifylline on oxygenation and exercise tolerance in COPD patients. 

The exact role which Pentoxifylline can play in COPD is still a subject for debate. We investigated the effects of Pentoxifylline in patients with severe to very severe COPD alongside pulmonary hypertension, using arterial oxygenation, the 6-Minute Walk Test (6MWT), and dyspnea score in this prospective, randomized, double-blind, placebo-controlled study. 

## Materials and Methods

The participants in this study were recruited from the Outpatient Pulmonary Clinic at Shiraz Medical Center. The study was approved by the Ethics Committee of Shiraz University of Medical Sciences, and informed consent was obtained from all the individuals before their participation.

*Patient Selection*

A total of 37 clinically stable patients with severe to very severe COPD were recruited. Patients were selected for the study if they had forced expiratory volume in 1 second (FEV1) of less than 50% of their predicted value and systolic pulmonary artery pressure greater than 40 mm Hg by color Doppler echocardiography. Seven patients did not meet the echocardiographic inclusion criterion and were, therefore, excluded. Patients were excluded from the study if they had systolic blood pressure more than 180 mm Hg, diastolic blood pressure more than 120 mm Hg, evidence of left ventricular dysfunction or symptomatic coronary artery disease, inability to walk for 6 minutes due to musculoskeletal disorders, significant exertional dysrhythmias, or symptomatic peripheral vascular disease. 

**Figure 1:**
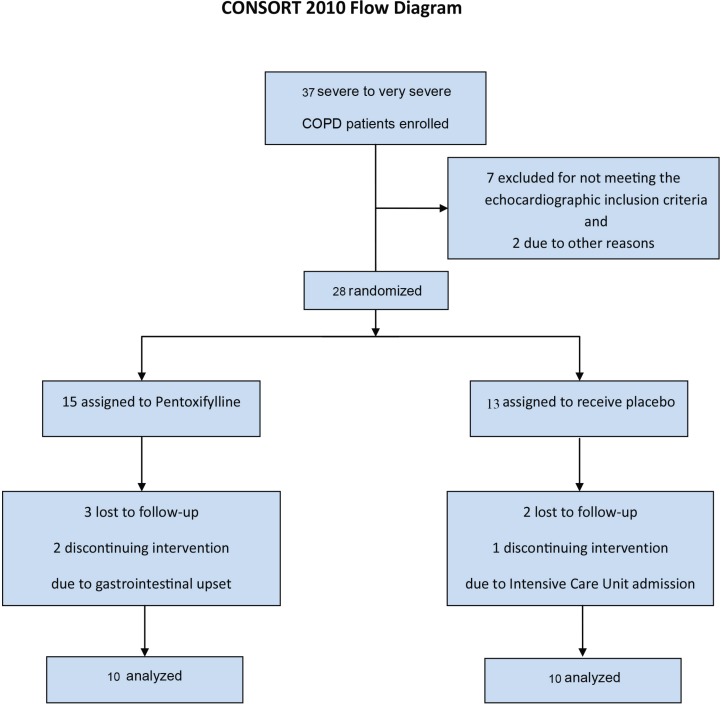
Enrollment and Outcomes.

Twenty-eight patients underwent randomization to receive either Pentoxifylline or placebo (figure 1). Three patients in the Pentoxifylline group and 2 in the placebo group were lost to follow-up. Given that there are only a few studies available in the existing literature on the effects of Pentoxifylline on COPD, we selected the number of our patients slightly higher than that of the previous studies. The patients were randomized via a simple method. Each patient received a drug package and his or her data were recorded in a questionnaire labeled with a randomly allocated number identical to that of the drug package through the study. 

The patients who qualified for the study underwent baseline spirometry and 6MWT in tandem with pulse oximetry and dyspnea rating before and after exercise with a standard Borg score questionnaire. Two patients in the case group stopped taking Pentoxifylline due to gastric complaints, and one patient in the placebo group withdrew from the study, after ICU admission due to COPD exacerbation. Finally, 10 patients in each group were analyzed.

Two trained nurses performed the 6MWT, pulse oximetry, and other measurements. The referring physicians, nurses, and patients were unaware of the contents of the drug package. The patients received either 400 mg of Pentoxifylline orally three times a day for 12 weeks or an identical-appearing placebo tablet with exactly the same dosing regimen. The Pentoxifylline or placebo dosage was halved in the patients receiving Theophylline. At 6 and 12 weeks post-intervention, the patients were re-evaluated.

*Statistical Analysis*

The SPSS (version 15) computer program was utilized for data entry and statistical analysis. The data were analyzed using the *t* test for mean comparisons, and the repeated measures ANOVA was employed to compare the differences between the two groups over the study period. All the measurements are expressed as mean±SD. A P value≤0.05 was considered significant.

## Results

The study population comprised 23 patients, of whom 12 received Pentoxifylline and 11 received the placebo (table 1). One patient in the placebo group (due to COPD exacerbation necessitating intensive care) and 2 in the Pentoxifylline group (due to adverse events) discontinued the study. Overall, the treatment groups were well matched with respect to baseline characteristics (table 1). All the patients were in the Global Initiative for Obstructive Lung Disease (GOLD) class of severe or very severe at baseline.

**Table 1:** Demographic and hemodynamic characteristics at baseline

	**Drug**	**Placebo**	**P value**
Male	12	9	-
Age (years)	64.6±8.4	66.6±12.5	0.67
FEV1* (ml)	990+177	930±369	0.60
SPAP** (mm hg)	47.8±7	48.9±6.67	0.60
6MWD*** (meters)	351.9±65	328±79.9	0.48
Estimated Vo_2 _peak (ml/kg/min)	13	12.5	0.48
Borg score (rest)	1.85±1.05	2.1±0.87	0.57
Borg score (post walk)	4±2.6	3.7±1.33	0.75
O_2_ saturation%**** (rest)	87±4	88±3	0.64
O_2_ saturation % (post walk)	85±8	83±6	0.73
Heart rate (/min) (rest)	81±11	89±9	0.15
Heart rate (/min) (post walk)	90±17	101±18	0.40

*Forced expiratory volume in 1 second; **Systolic pulmonary artery pressure; ***Six-minute walk distance;****By pulse oximetry

**Figure 2:**
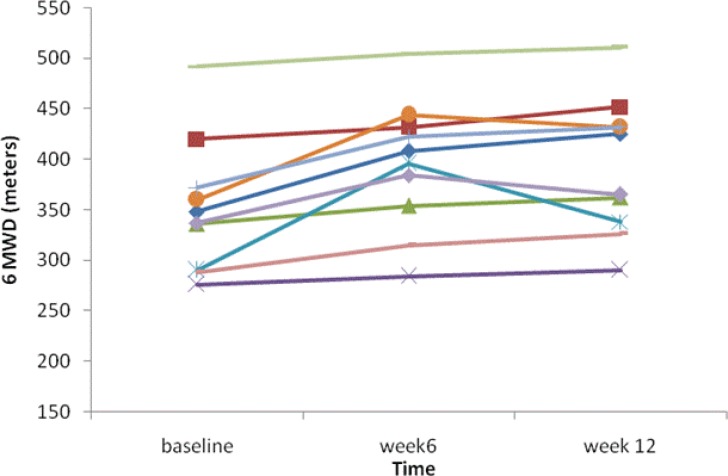
Individual 6-minute walk distance (MWD) in the Pentoxifylline group is plotted against time in weeks.

**Figure 3:**
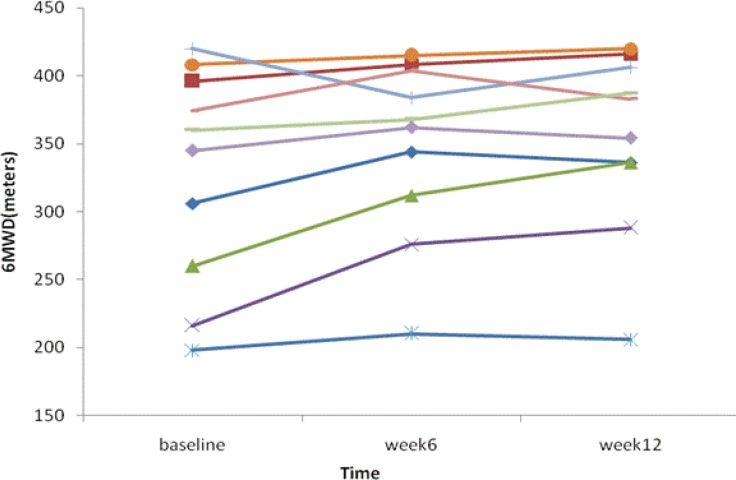
Individual 6-minute walk distance (MWD) in the placebo group is plotted against time in weeks.

The mean 6-minute walk distance increased by 41 meters in the Pentoxifylline group (351.9±65 at baseline to 393±67 meters at week 12; P<0.001), and increased by 25 meters in the placebo group (328±79 at baseline to 353±66 meters at week 12; P<0.001). Despite the significant increase in the 6-minute walk distance in both groups, there was no statistically significant difference between the groups (P=0.142). After the administration of Pentoxifylline for 12 weeks, there was no increase (compared to the placebo) in the mean resting arterial oxygen saturation and heart rate, or nor was there a decrease in dyspnea score (table 2). The individual 6-minute walk distance of both patient groups is plotted against time in figures 2 and 3.

**Table 2:** Changes in 6-Minute Walk Test, dyspnea score, and oxygenation before and after Pentoxifylline administration

**Variable**	**Week 0**		**Week 6**		**Week 12**		**P value**
	**Drug**	**Placebo**	**Drug**	**Placebo**	**Drug**	**Placebo**	
6 MWD * (meters)	351.9±65	328±79	394±64	348±65	393±67	353±66	0.142
Estimates Vo_2_ peak (ml/kg/min)	13	12.5	14	13	14	13	0.294
Borg score (pre test)	1.8±1	2.2±0.8	1.2±0.9	2.2±0.9	1.1±1.3	1.9±0.9	0.126
Borg scale (post test)	4±2.6	3.7±1.3	3.3±2.3	3.7±1.6	3.5±2.7	3.6±1.5	0.539
O_2_ saturation% (pre test)	87±4	88±3	88±4	88±3	85±14	88±2	0.676
O_2_ saturation% (posttest)	85±8	83±6	87±6	86±5	87±6	86±4	0.818
Pulse rate/min (pre test)	81±11	89±9	82±13	86±7	80±11	88±7	0.582
Pulse rate /min (post test)	90±17	101±18	96±18	100±11	93±20	101±9	0.616

*Six-minute walk distance

## Discussion

COPD is characterized by dyspnea-induced impairment and as such can significantly limit the performance of everyday tasks. Hence, a primary goal in the management of COPD is to improve dyspnea with a view to facilitating physical activities irrespective of the severity of the disease if the patient’s health-related quality of life is to be enhanced.^20^

Pentoxifylline is a xanthine-derived agent, which possesses several properties that could have beneficial effects for the patient with COPD. It improves the flow properties of blood by decreasing blood viscosity and reducing RBCs and platelet aggregation.^21^ It also increases cardiac output and O2 consumption and attenuates systemic vasoconstriction.^22^ The drug is currently used in patients with peripheral vascular disease to increase blood perfusion and improve oxygen delivery. In addition, Pentoxifylline has been reported to increase the cardiac index and there is preliminary evidence that it can reduce hypoxia-induced pulmonary vasoconstriction.^6^

In the current study, the hypothesis that the net effect of this constellation of pharmacologic properties would improve gas exchange in COPD patients was tested in a group of patients with severe and very severe COPD in conjunction with pulmonary hypertension immediately after exercise. Haas et al.^3^ demonstrated that Pentoxifylline improved treadmill walk time, arterial saturation, and pulmonary gas exchange in patients with moderate to severe COPD. Why did we obtain such disparate results relative to that study? There are a number of possible explanations. First, there are methodological differences between the two studies. Our study was double-blinded and placebo-controlled, which lessened any bias that might have occurred during the exercise testing. We also compared our exercise indices after the administration of Pentoxifylline for 12 weeks to the indices after the administration of the placebo for 12 weeks, whereas the previous investigators compared the exercise indices after Pentoxifylline administration with their baseline indices. Second, the patient populations may have been slightly different. In the study by Haas et al.^3^ the patients who received Pentoxifylline had a mean Hb saturation of oxygen of 92.8% with a range of 89 to 96%, while in our study, the mean Hb saturation of oxygen was 87.5% with a range of 83 to 91% with pulmonary hypertension; consequently, failure of Pentoxifylline to improve oxygenation and 6-minute walk distance in our COPD patients may have been caused by the recruitment of previously unrecruited capillaries as a result of hypoxemia. All the COPD patients in the study by Haas et al.^3^ who received Pentoxifylline for 12 weeks, had ceased smoking a minimum of five years earlier; while all our patients were smokers at the time of examination. In agreement with our results, Scott et al.^19^ in a double-blind study did not find evidence of improvement in oxygenation, resting diffusion capacity of lung for carbon monoxide (DLco), exercise tolerance, and dyspnea after a 12-week course of Pentoxifylline in individuals with moderate to severe COPD. Finally, the patients included in this trial had pulmonary hypertension at rest. Therefore, the current results are only generalizable to patients with severe COPD in tandem with mild to moderate pulmonary hypertension at rest and may not as such apply to patients with less severe COPD. In this context, these findings do not rule out the notion that Pentoxifylline may be efficacious in patients with less severe airflow obstruction.

## Conclusion

Despite the potential advantageous properties of Pentoxifylline in this randomized, double-blind, placebo-controlled study on 23 patients with severe to very severe COPD receiving Pentoxifylline for 12 weeks, we found no improvement in oxygenation, 6MWT, or dyspnea score.
